# Synthetic Document Images with Diverse Shadows for Deep Shadow Removal Networks

**DOI:** 10.3390/s24020654

**Published:** 2024-01-19

**Authors:** Yuhi Matsuo, Yoshimitsu Aoki

**Affiliations:** Department of Electrical Engineering, Faculty of Science and Technology, Keio University, 3-14-1 Hiyoshi, Kohoku-ku, Yokohama 223-8522, Kanagawa, Japan; aoki@elec.keio.ac.jp

**Keywords:** shadow removal, document images, deep neural networks

## Abstract

Shadow removal for document images is an essential task for digitized document applications. Recent shadow removal models have been trained on pairs of shadow images and shadow-free images. However, obtaining a large, diverse dataset for document shadow removal takes time and effort. Thus, only small real datasets are available. Graphic renderers have been used to synthesize shadows to create relatively large datasets. However, the limited number of unique documents and the limited lighting environments adversely affect the network performance. This paper presents a large-scale, diverse dataset called the Synthetic Document with Diverse Shadows (SynDocDS) dataset. The SynDocDS comprises rendered images with diverse shadows augmented by a physics-based illumination model, which can be utilized to obtain a more robust and high-performance deep shadow removal network. In this paper, we further propose a Dual Shadow Fusion Network (DSFN). Unlike natural images, document images often have constant background colors requiring a high understanding of global color features for training a deep shadow removal network. The DSFN has a high global color comprehension and understanding of shadow regions and merges shadow attentions and features efficiently. We conduct experiments on three publicly available datasets, the OSR, Kligler’s, and Jung’s datasets, to validate our proposed method’s effectiveness. In comparison to training on existing synthetic datasets, our model training on the SynDocDS dataset achieves an enhancement in the PSNR and SSIM, increasing them from 23.00 dB to 25.70 dB and 0.959 to 0.971 on average. In addition, the experiments demonstrated that our DSFN clearly outperformed other networks across multiple metrics, including the PSNR, the SSIM, and its impact on OCR performance.

## 1. Introduction

With the popularization of high-performance cameras in smartphones, many people have started using phone cameras instead of scanners to digitize documents. Compared to scanners, however, capturing documents with a camera often leads to problems such as shadows, because light sources might be occluded by the camera or the user’s hand. Even without occluders, uneven illumination is likely to occur. Removing shadows from document images is an essential task because shadows and uneven illumination hinder legibility for users and affect the performance of various computer vision tasks, such as optical character recognition (OCR).

Most traditional document shadow removal methods use heuristics to explore document image characteristics [1,2,3,4,5]. However, owing to limitations of the heuristics, these approaches will often only work well for some document images but fail for others [6]. Deep learning-based methods have recently been applied to various computer vision and document shadow removal tasks, demonstrating promising results. Many shadow removal methods train on given sets of shadow images and shadow-free images to remove shadows in an end-to-end manner [6,7,8,9]. Existing studies on shadow removal from a single document have proposed real datasets containing pairs of shadow and shadow-free document images. However, small real datasets had been available due to the cost of creating large datasets [1,2,3,5,6]. To combat this problem, comparatively large datasets are created that have real-world document pairs with various samples under different lighting conditions [10,11]. However, it is still difficult to produce data with a comprehensive variety of characteristics in a real environment. Lin et al [6] also create a relatively large dataset by synthesizing shadows to documents using a graphic renderer. Nonetheless, the lighting environments, including various occluders and environment maps, are limited. The small number of unique documents also remains a limiting factor to the trained network’s performance.

We address these issues by creating a synthetic dataset that alleviates the limitations on the number and diversity of data, which allows the deep shadow removal network to perform better and more robustly for various characteristics. This paper builds a large-scale, diverse synthetic dataset rendered in various environments with abundant materials using a graphic renderer, dubbed Synthetic Document with Diverse Shadows (SynDocDS). They are further diversified based on our observations and the shadow synthesis pipeline [12], which considers shadow characteristics to obtain more plausible data. Furthermore, in shadow removal, it is crucial for the network to specifically learn the location of the shadows. Moreover, inspired by Bako et al. [1], it can be assumed that document images often have constant background colors; thus, the network for document shadow removal requires a high understanding of global color features. This paper proposes a network that removes shadows from a document image with high global color comprehension and learns shadow regions. We call our proposed network the Dual Shadow Fusion Network (DSFN). Experiments showed that the deep shadow removal networks trained only on the proposed SynDocDS dataset performed well on real data, and performance improvements were seen when the SynDocDS dataset was used for pre-training. Finally, we demonstrated that the proposed DSFN yielded better results than other methods.

Our contributions are as follows:

- We propose the synthetic dataset called SynDocDS, a large-scale, diverse synthetic document dataset comprising shadow images, shadow-free images, and shadow mattes in various scenes. The dataset is diversified based on our observations regarding the illumination model. The source code and datasets will be released.
- We show that (pre-)training on the SynDocDS dataset results in more effective and robust networks than training on a limited real dataset.
- We propose a new network for shadow removal that fuses multiple features and shadow attentions efficiently. Experimental results show that our network yields better results than other networks.

Section 2 surveys the works related to shadow removal and shadow synthesis. Following that, Section 3 introduces our novel dataset. Section 4 details the architecture of the proposed network for document shadow removal. In Section 5, the experimental results are presented, and Section 6 discusses the results.

## 2. Related Work

In this section, firstly, we review the general shadow removal method for natural images in Section 2.1. In addition, we survey the works related to document shadow removal. Secondly, we go over existing works on shadow synthesis for creating shadow removal datasets in Section 2.2.

### 2.1. Shadow Removal

**Natural images.** Some traditional methods use handcrafted features and achieved shadow removal using physical models of illumination and color [13,14]. However, their performance is limited. In recent years, several deep learning-based methods have been proposed for shadow removal in natural images, achieving state-of-the-art performances. Deep learning-based methods achieve the removal of shadows by learning complex mappings on large datasets containing shadow images, shadow-free images, and shadow masks [15,16]. STCGAN [7] trains to perform shadow detection and removal simultaneously by stacking two conditional GANs. A directionally aware method has been proposed to obtain 2D spatial context from four directions [8]. Moreover, the work by Fu et al. [17] adaptively fuses multiple estimated overexposed images using a shadow-aware fusion network to generate shadow-free images. DHAN [9] incorporates spatial attention and learns the shadow regions explicitly in a hierarchical layer aggregation style [18]. MaskshadowGAN [19] and LG-ShadowNet [20] exploit GAN-based models to perform unsupervised shadow removal by learning maps between shadow and unshadowed regions. Liu et al. [21] propose a shadow generation model to construct pairs of pseudo-shadow and shadow-free images for weakly supervised shadow removal. Methods that do not require paired teacher images have also been introduced for shadow removal for natural images in recent years. However, un/self/weakly-supervised learning is generally not able to achieve a performance superior to supervised learning with paired data [19,20,21].

**Document images.** Several methods specifically designed to remove shadows from document images have been proposed. The water-filling method by Jung et al. [3] converts the input image into a topographic surface and simulates an immersion process. However, it tends to apply a color shift, resulting in brighter colors compared to the original. Kligler et al. [2] try to improve the quality of document images by representing an image as a 3D point cloud and selecting pixels to be restored using the visibility detection method. However, the results often contain shadow edges. Bako et al. [1] calculates the ratio of the global background color to the local background color of each patch to obtain a shadow map and adjusts the shadows of the input image according to the shadow map. Since these methods detect the background area and interpolate the remainder, they fail if the document contains a large area of figures and shadows. Lin et al. [6] are the first to propose a deep learning method for document shadow removal, achieving promising results. Their method uses the estimated background color and an attention map obtained by GradCAM [22]. Zhang et al. [10] propose a method for extracting a background image that accurately depicts the background colors. They also propose a network that uses the predicted spatially varying background as auxiliary information. Li et al. [11] design a shadow removal network that can effectively learn low-frequency details and high-frequency boundaries for high-resolution document images.

### 2.2. Shadow Synthesis

As mentioned in the previous section, many deep learning-based shadow removal methods have been proposed in recent years. These methods learn based on a set of shadow and shadow-free image pairs. These general supervised learning methods require a large set of paired images. However, due to the considerable cost of creating such datasets, currently available real image datasets for document shadow removal are small and are mainly intended for evaluation purposes [1,2,3,5,6]. Table 1 summarizes the shadow removal datasets for document images. Training on such limited data significantly affects the network’s performance because the network cannot fully understand the scene [9]. Therefore, existing studies have proposed training deep learning models by generating pseudo-images.

Several methods exist to synthesize shadow images for shadow removal. Some of the approaches directly render the shadow/shadow-free image pairs with a 3D renderer [23,24]. In the work by Sidrov [23], pairs are created in an urban landscape in a computer game. In [24], shadows are projected on a plane with a texture of realistic images on the surface using ray tracing in Maya [25]. The pipeline by Gryka et al. [24] is limited because the settings only contain a single light source (in addition to the simulated sky and global illumination) and a single occluder. Furthermore, the resulting rendering is not plausible since the material information of the texture image is not accessible. Other methods for synthesizing realistic shadows have recently been proposed using GANs. For example, the Shadow Matting GAN (SMGAN) [9] synthesizes a shadow image by taking a shadow-free image and a randomly sampled shadow mask. However, since the SMGAN learns from existing datasets, the performance is severely limited when the training data are small and biased. Thus, deep learning-based methods are still necessary and require training data closer to the real world, whereas having paired data is desirable. SynShadow [12] extends a physics-based illumination model inspired by [14,15] rather than rendering shadows directly using a renderer. A shadow is synthesized into an arbitrary shadow-free image using a shadow matte by randomizing the parameters of the shadow model. SynShadow can produce infinite combinations of shadow mattes and shadow-free images and generate hundreds of shadows with different intensities, even for the same pair. However, the shadow mattes assume a flat plane, and the synthesized shadows may not perfectly match the geometry of the background. In addition, the sampling distributions of the parameters are determined only from observations of natural images (ISTD+ [7,15], SRD [16]),which were taken outside. For images with significantly different characteristics (e.g., an indoor environment), selecting appropriate parameter distributions becomes necessary again.

In this way, synthetic images are often used to train deep neural networks for shadow removal. Similarly, synthetic data have been proposed for document shadow removal. DocIIW [26] provides a sizeable multi-illuminated document dataset, Doc3DShade [26], that extends the public dataset Doc3D [27]. This study uses randomly distorted paper to capture shadows under complex lighting conditions. Furthermore, using various types of diffuse paper materials, such as magazines, newspapers, and printed documents, they considered the material properties of the shadows under complex lighting conditions that are impossible with rendering engines. However, the shadows contained in the Doc3DShade dataset are caused by geometric shapes, and their characteristics differ significantly from shadows created when an occluder blocks primary light. Therefore, they are not applicable for removing hard shadows caused by such conditions. In the work by Lin et al. [6], a comparatively large dataset called the Synthetic Document Shadow Removal Dataset (SDSRD) has been proposed, in which shadows are synthesized on captured images, mostly from the PRImA Layout Analysis dataset [28], using Blender [29]. This allows us to use a large variety of document images. However, the number of unique documents is limited to about 1000, while the diversity of the generated images is minimal due to the limited variety of occluders and environment maps used for rendering. In addition, as in [24], the lack of information about materials and consideration of paper geometry limits the network’s performance. In this paper, we observe document characteristics more closely and propose a significantly more diverse and large-scale synthetic dataset for document shadow removal.

## 3. Synthetic Documents with Diverse Shadows

**Figure 1:**
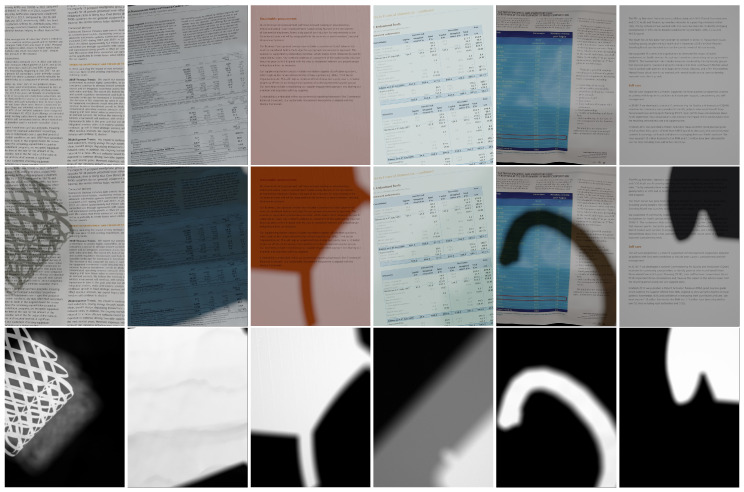
Example triplets from SynDocDS dataset. From top to bottom: shadow-free images, shadow images, and shadow mattes.

For document shadow removal, we propose a new diverse dataset, SynDocDS. Examples are shown in Figure 1. The process of creating the SynDocDS dataset comprises two steps: (i) image rendering and (ii) shadow augmentation. We describe both steps in detail in this section.

### 3.1. Image Rendering

**Figure 2:**
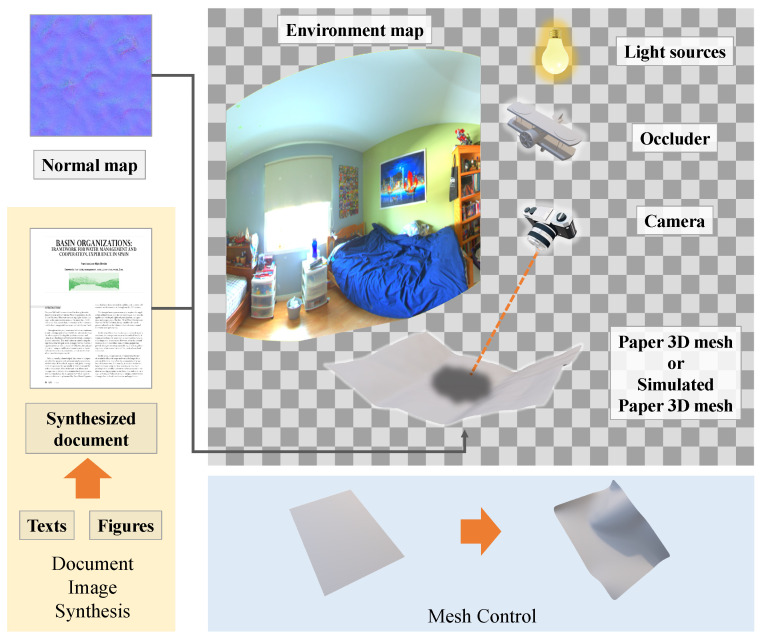
Overview of rendering.

First, we describe the rendering settings. Synthetic document images are obtained by creating documents from text and figures and rendering images with and without shadows using path tracing [30] in rendering software, Blender (2.82a) [29], under diverse conditions. In addition, only the shadow regions can be extracted using Blender’s function, which is called Shadow Catcher, and we use these images as a shadow matte. Figure 2 shows an overview of the rendering settings. The details of each rendering setting are described below, corresponding to the elements written in Figure 2.

**Document image synthesis.** First, text data and image data from the DDI-100 dataset [31] are combined to create document images. When compositing, the text and images are positioned so they do not overlap. Note that although the text data in DDI-100 are provided as a binary image, it is possible to use standard text by adding positional information. The textures of the synthesized document images are subsequently applied to the document mesh.

**Mesh control.** To extend the background document images, we introduce random geometric distortions to a paper mesh, applying pressure from around the edges to the center of it by considering the physical properties. Detailed mesh properties are shown in Table 2. Additionally, we use distorted paper meshes from Doc3D [27], the 3D dataset with realistic paper for warping and renderings. Using a mesh of documents manipulated in this way dramatically increases the diversity of the dataset. During rendering, the mesh is randomly sampled, while the probability of plane and distorted mesh is set to equal.

**Normal map.** Fine details, such as wrinkles on the paper’s surface, are represented by applying a normal map to the document mesh. We use the randomly sampled sand, and fabric normal maps from the SVBRDFs dataset [32].

**Occluder.** We adopt ShapeNet [33] for occluders, a publicly available 3D model dataset. Then, we randomly sample a single 3D mesh from ShapeNet and use the geometric information while rendering a single shadow document image.

**Environment map.** To enrich the background scene, we use SUN360 [34] and the Laval Indoor HDR dataset [35], which provides environment maps in the form of panoramas. Although SUN360 [34] contains indoor/outdoor panoramas, the Laval Indoor HDR dataset [35] only contains indoor panoramas. We randomly sample panoramas but ensure the indoor and outdoor frequencies are the same.

**Light sources.** To increase the diversity of the dataset, the rendering is performed with different combinations of the number/radius of lights and colors. The number of lights ranges from 1 to 4. The lights are placed 2.2 m away from the paper and the radius is randomly determined in the range of [0.01, 0.05]. The color of light is determined by uniformly sampling the hue and saturation according to [0, 1] and [0, 0.3] from a range normalized to 0–1, respectively. The values are fixed at 1.

**Camera.** The virtual paper is captured through the camera as shown in Figure 2. The camera is positioned 1.5 m above the paper, and the lens principal point is set to be the center of the paper. All occluder objects are also placed outside the camera view.

### 3.2. Enriching Shadow Images

To further diversify and enrich the rendered shadow images, the shadow images are augmented following SynShadow [12], which considers shadow characteristics. In the work by Shor et al. [14], following the image formation equation [36], and assuming that the affine nature of the relationship between illuminated and shadowed intensities does not change, the relation between $I^{\mathrm{lit}}$ and $I^{\mathrm{dark}}$ at any pixel is formulated as follows:

(1) $$\begin{array}{c} I_{k}^{\mathrm{lit}}=\alpha_{k}+\gamma I_{k}^{\mathrm{dark}}, \end{array}$$

where $\alpha_{k},k\in \left\{0,1,2\right\}$ represents the camera’s spectral response to the reflected direct illumination in the RGB color channels and γ is the inverse of the ambient attenuation factor. Both $\alpha_{k}$ and γ are scalar values. Based on the above equation, a shadow synthesis pipeline was proposed by Inoue and Yamazaki [12]. To compute a dark image $I_{ijk}^{\mathrm{dark}}$ , where all the pixels are shadowed and have the same attenuation property, from shadow-free image $I_{ijk}^{\mathrm{sf}}$ , the affine model in Equation (1) yields the following:

(2) $$\begin{array}{c} I_{k}^{\mathrm{dark}}=\frac{1}{\gamma }I_{k}^{\mathrm{sf}}-\frac{\alpha_{k}}{\gamma }. \end{array}$$

**Figure 3:**
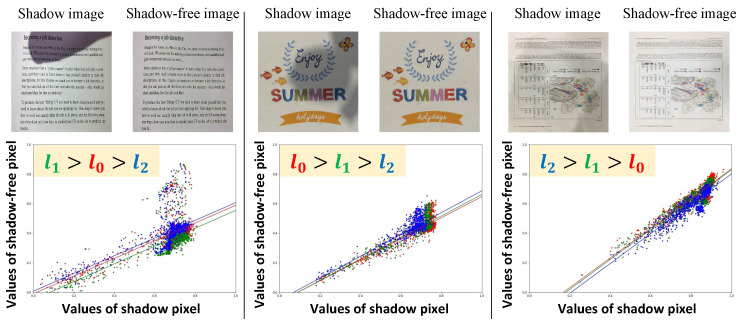
Plots of the shadow attenuation for each of the three color channels in the document images. The horizontal axis corresponds to the value of shadow images and the vertical axis to the shadow-free images. Left to right, the examples are from the OSR [5], Kligler’s [2], and Jung’s datasets [3], respectively.

In [12] $,\alpha_{k}$ and γ are converted to four parameters $\left(l_{0},l_{1},l_{2},s1\right)$ , which are written as $s_{1}=\frac{1-\alpha_{k}}{\gamma }$ , $l_{k}=\alpha_{k}$ . Then, Equation (2) is explained as follows:

(3) $$I_{ijk}^{\mathrm{dark}}=\left\{\begin{array}{cc} \frac{s_{1}}{1-l_{1}}\left(I_{ijk}^{\mathrm{sf}}-l_{k}\right)\;\; & \mathrm{if}\;\;I_{ijk}^{\mathrm{sf}}-l_{k}\geq 0,\\ 0\;\; & \mathrm{if}\;\;I_{ijk}^{\mathrm{sf}}-l_{k}<0, \end{array}\right.$$

where *i* and *j* are pixel indices. To obtain a plausible range of shadows, in [12], the set of parameters is determined based on the observation that $\left(l_{0},l_{1},l_{2}\right)$ are correlated. The relation is often $l_{0}>l_{1}>l_{2}$ due to the blueish ambient light from the sky in outdoor scenes. However, the observation is based on ISTD+ [7] and SRD [16] and is not optimal for synthetic shadow document image generation. Therefore, we visualize the shadow attenuation of each RGB channel, following [12,14], for an existing dataset of document images from the validation set (these images are not used during testing), as shown in Figure 3. In contrast to the observation in [12], we found that the magnitude relation between $\left(l_{0},l_{1},l_{2}\right)$ varies depending on the characteristics of the image, even in the same dataset. For example, the indoor environment is affected by various ambient light sources, unlike outdoor photography. Since the distribution of each parameter is different, in this study, the parameter settings are determined by considering real document image datasets as illustrated in Figure 3. We introduce $\Delta l_{0}=l_{0}-l_{1}$ , and $\Delta l_{2}=l_{2}-l_{1}$ and sample $\left(l_{1},s_{1},\Delta l_{0},\Delta l_{2}\right)$ . Both $l_{1}$ and $s_{1}$ follow a uniform distribution U(a,b) . We employ (a,b)=(0.1,0.125) and (a,b)=(0.1,0.9) for $l_{1}$ and $s_{1}$ , respectively. Both $\Delta l_{0}$ and $\Delta l_{2}$ follow normal distribution N(μ,σ) . We employ (μ,σ)=(0,0.03) for $\Delta l_{0}$ and $\Delta l_{2}$ .

Finally, the shadow image $I_{k}^{s}$ is obtained by alpha composing $I_{k}^{\mathrm{sf}}$ and $I_{k}^{\mathrm{dark}}$ using the shadow matte *M* as the alpha factor:

(4) $$\begin{array}{c} I_{k}^{s}=\left(1-M\right)⊙I_{k}^{\mathrm{sf}}+M⊙I_{k}^{\mathrm{dark}}. \end{array}$$

**Figure 4:**
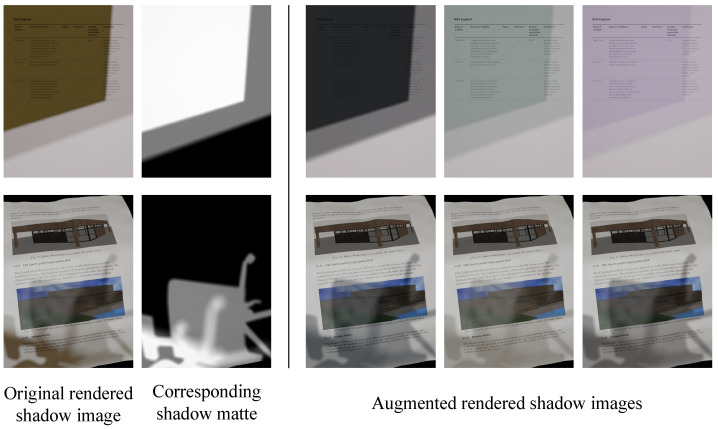
Examples of augmented rendered shadow images.

As opposed to [12], we achieve more realistic shadow images as the composited shadow fully matches the background geometry. We show examples in Figure 4. We use this synthesis pipeline to extend the rendered shadow images. In this study, the number of rendered images was extended by a factor of 10.

## 4. Method

**Figure 5:**
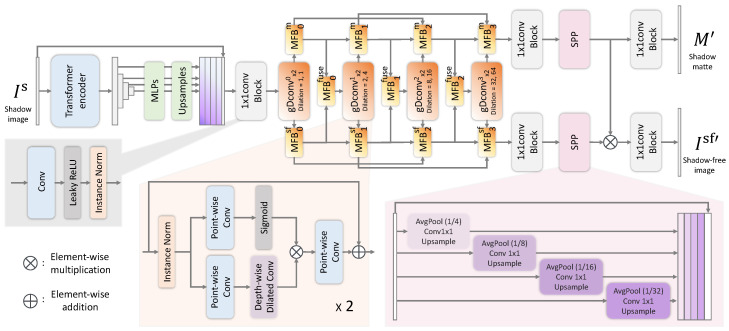
The network architecture of the proposed Dual Shadow Fusion Network.

This paper proposes the Dual Shadow Fusion Network (DSFN) to remove shadows from a document image. In Figure 5, we illustrate the overall architecture of the DSFN. To train our network, we use N triplets $\left\{I^{s},I^{\mathrm{sf}},M\right\}$ , each comprising a shadow image $I^{s}$ , a shadow-free image $I^{\mathrm{sf}}$ , and a shadow matte *M*. Given $I^{s}$ , the proposed network learns the shadow-free image $I^{\mathrm{sf}}$ with the help of the attention loss using *M*. The DSFN actually outputs the predicted shadow-free image ${I^{\mathrm{sf}}}^{\prime }$ , and the shadow matte $M^{\prime }$ : $\left({I^{\mathrm{sf}}}^{\prime },M^{\prime }\right)=\;DSFN\left(I^{s}\right)$ . In this section, we detail the network structure of the proposed DSFN in Section 4.1. Additionally, we describe the loss functions and training settings in Section 4.2.

### 4.1. Dual Shadow Fusion Network

For shadow removal, it is crucial for the network to specifically learn the location of the shadows. In addition, document images, unlike ordinary images, often have constant background colors, which requires networks to develop an understanding of global color features. Our DSFN is mainly based on the Dual Hierarchical Aggregation Network (DHAN) [9] for removing shadows from natural images. The DHAN [9] incorporates an attention module for spatial attentions and mixed layer features. The network learns the shadow regions explicitly in a hierarchical layer aggregation style [18]. First, to extract rich representation, we used parts of the SegFormer architecture [37] as a backbone. SegFormer comprises a novel hierarchical transformer encoder that outputs multiscale features and a multilayer perceptron (MLP) decoder that aggregates information from different layers. This achieves powerful representations combining local and global attention [37], leading to a high understanding of global color [38]. The transformer encoder is pre-trained on ImageNet-1k [39] as in [37]. Unlike the original SegFormer paper [37], the last MLP is not used, and the feature map is resized to the same height and width as the input in the upsample layer. The encoded features are then concatenated with the input image and input to the 1 × 1 convolutional layer. In the next step, we encode multiscale features from the transformer using several dilated convolutions. To learn the shadow regions more specifically, we adopt a gating mechanism proposed in [40], which acts as the pixel attention module and nonlinear activation function, with dilated convolution called gDconv, as shown in Figure 5. To aggregate features hierarchically and merge shadow attention and features, we propose a Multifusion Block (MFB) that fuses the multiple feature maps from different paths dynamically. Finally, we used a spatial pooling pyramid [41] after the last MFB for feature mixing.

**gDconv.** Our gDConv is mainly based on a gating mechanism proposed in [40] and used as stacked two layers. The *n*-th $gDconv_{x2}^{n}$ layer is defined as follows:

(5) $$gDConv_{\times 2}^{n}\left(x\right)=\left\{\begin{array}{cc} gDConv_{1}\left(gDConv_{1}\left(x\right)\right)\;\; & \mathrm{if}\;\;n=0,\\ gDConv_{2^{2n}}\left(gDConv_{2^{2n-1}}\left(x\right)\right)\;\; & otherwise. \end{array}\right.$$

Given feature maps *x*, the $gDconv_{k}$ normalizes them using InstanceNorm IN(·) . Then, we use a point-wise convolutional layer PWC(·) and depth-wise dilated convolutional layer DWDC(·) . The feature applied with the sigmoid function Sigmoid(·) is used as the gating signal and is summed with the identity input *x*, which can be formulated as follows:

(6) $$\begin{array}{c} gDConv_{k}\left(x\right)=PWC\left(Sigmoid\left(PWC\left(IN\left(x\right)\right)\right)\cdot DWDC_{k}\left(PWC\left(IN\left(x\right)\right)\right)\right)+x, \end{array}$$

where *k* represents the k-dilated convolution. These gating mechanisms act as the pixel attention module, which is sometimes used for in-painting tasks to restore partially degraded regions or for image restoration tasks to improve the capability of networks [42,43].

**Figure 6:**
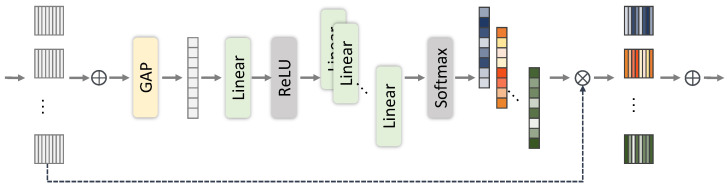
Multifusion Block (MFB).

**Multifusion Block.** Our Multifusion Block (MFB) is a modified multipath version of SK Fusion [40], a simplified SK module [44]. We show an overview of the MFB in Figure 6. The MFB takes multiple feature maps $\left\{x_{0},x_{1},\ldots ,x_{N}\right\}$ and then fuses them via $y=\sum_{i}^{N}a_{i}\cdot x_{i}=MFB\left(\left\{x_{0},x_{1},\ldots ,x_{N}\right\}\right)$ with fusion weights $a_{i}$ . To obtain the fusion weights, we use global average pooling GAP(·) , MLP (Linear-ReLU-Linear) MLP(·) , a softmax function F(·) , and a split operation:

(7) $$\begin{array}{c} \left\{a_{0},a_{1},\ldots ,a_{N}\right\}=Split\left(F\left(MLP\left(GAP\left(\sum_{i}^{N}x_{i}\right)\right)\right)\right). \end{array}$$

This MLP plays the role of reducing and increasing dimensionality, similar to the channel attention mechanism, which can re-weight each feature channel proposed in [45].

**Dual hierarchical aggregations.** Our proposed network is constructed in a hierarchical layer aggregation style based on [9,18] for spatial attentions and mixed layer features. Our DSFN merges the features from gDConv using MFBs for attention. The features obtained from each layer can be defined as follows:

(8) $$\begin{array}{c} x_{n}^{fuse}=MFB_{n}^{fuse}\left(\left\{gDConv_{\times 2}^{n}\left(x_{n}^{m}\right),gDConv_{\times 2}^{n}\left(x_{n}^{sf}\right)\right\}\right), \end{array}$$

where *n* represents the *n*-th layer. The shadow attention and shadow-free features, $x^{m}$ and $x^{sf}$ , are defined as follows:

(9) $$x_{n}^{m}=\left\{\begin{array}{cc} MFB_{n}^{m}\left(\left\{gDConv_{\times 2}^{n}\left(\hat{x}\right)\right\}\right)\;\; & \mathrm{if}\;\;n=0,\\ MFB_{n}^{m}\left(\left\{x_{n-1}^{m},gDConv_{\times 2}^{n}\left(x_{n-1}^{fuse}\right)\right\}\right)\;\; & \mathrm{if}\;\;n=1,\\ MFB_{n}^{m}\left(\left\{x_{n-2}^{m},x_{n-1}^{m},gDConv_{\times 2}^{n}\left(x_{n-1}^{fuse}\right)\right\}\right)\;\; & otherwise, \end{array}\right.$$

(10) $$x_{n}^{sf}=\left\{\begin{array}{cc} MFB_{n}^{sf}\left(\left\{gDConv_{\times 2}^{n}\left(\hat{x}\right)\right\}\right)\;\; & \mathrm{if}\;\;n=0,\\ MFB_{n}^{sf}\left(\left\{x_{n-1}^{sf},gDConv_{\times 2}^{n}\left(x_{n-1}^{fuse}\right)\right\}\right)\;\; & \mathrm{if}\;\;n=1,\\ MFB_{n}^{sf}\left(\left\{x_{n-2}^{sf},x_{n-1}^{sf},gDConv_{\times 2}^{n}\left(x_{n-1}^{fuse}\right)\right\}\right)\;\; & otherwise, \end{array}\right.$$

where $\hat{x}$ is the encoded features obtained from the SegFormer encoder [37]:

(11) $$\begin{array}{c} \hat{x}=Conv_{1\times 1}\left(Cat\left(\left\{SegFormerEnc\left(I^{s}\right),I^{s}\right\}\right)\right). \end{array}$$

Cat(·) means the feature concatenation. For each feature obtained from the last layer, a spatial pooling pyramid (SPP) [41] is applied to fuse multilevel features:

(12) $$\begin{array}{cc} {I^{\mathrm{sf}}}^{\prime } & =Conv_{1\times 1}(Sigmoid\left(SPP\left(x_{n}^{m}\right)\cdot SPP\left(x_{n}^{sf}\right)\right),\\ M^{\prime } & =Conv_{1\times 1}(Sigmoid\left(SPP\left(x_{n}^{m}\right)\right). \end{array}$$

The Sigmoid(·) layer is added only at the end of the shadow attention. Therefore, our DSFN outputs the predicted shadow-free image ${I^{\mathrm{sf}}}^{\prime }$ , and the shadow matte $M^{\prime }$ : $\left({I^{\mathrm{sf}}}^{\prime },M^{\prime }\right)=DSFN\left(I^{s}\right)$ .

### 4.2. Loss Functions

The basic loss function is L1 loss, which is defined as the absolute error between the ground truth $I^{\mathrm{sf}}$ and the output ${I^{\mathrm{sf}}}^{\prime }$ .

(13) $$\begin{array}{c} L_{r}=\sum_{i=1}^{N}{\left\|I^{\mathrm{sf}}-{I^{\mathrm{sf}}}^{\prime }\right\|}_{1}. \end{array}$$

Since the output from convolutional neural networks might leave artifacts around the shadow edges, degrading the quality of shadow removal as mentioned in [9], we use the adversarial loss [46]. The proposed network can be considered the generator, while the discriminator D comprises five convolutional layers, a ReLU, and Batch Normalization following [6,7,9]. The ground truth $I^{\mathrm{sf}}$ and the predicted shadow-free image ${I^{\mathrm{sf}}}^{\prime }$ are discriminated by patch. The generator outputs a realistic image to fool the discriminator, and the discriminator is optimized to identify the generated image. The loss is as follows:

(14) $$\begin{array}{c} L_{cGAN}=logD\left(I^{s},I^{\mathrm{sf}}\right)+log\left(1-D\left(I^{s},{I^{\mathrm{sf}}}^{\prime }\right)\right). \end{array}$$

Furthermore, we use the perceptual loss [47] to account for semantic measures and low-level details in multiple contexts. This utilizes a convolutional neural network Φ that has been pre-trained for image classification. In this research, Φ is a 19-layer VGG network [48] pre-trained on the ImageNet dataset [39]. Let $\Phi_{i}\left(x\right)$ be the feature map obtained from the *i*-th activation layer of the network when processing image *x*. We use layers 1 to 5 and define the perceptual loss as follows:

(15) $$\begin{array}{cc} \; & L_{p}=\sum_{i=1}^{N}{\left\|\Phi_{i}\left(I^{\mathrm{sf}}\right)-\Phi_{i}\left({I^{\mathrm{sf}}}^{\prime }\right)\right\|}_{1}. \end{array}$$

The L1 loss between the predicted shadow matte $M^{\prime }$ and the ground truth shadow matte *M* is used for learning the shadow regions, as the shadow matte *M* is a gray-scale image with continuous values:

(16) $$\begin{array}{c} L_{s}=\sum_{i=1}^{N}{\left\|M-M^{\prime }\right\|}_{1}. \end{array}$$

The final objective function is therefore as follows:

(17) $$\begin{array}{c} \underset{G}{min}\underset{D}{max}\;\lambda_{0}L_{cGAN}+\lambda_{1}L_{r}+\lambda_{2}L_{p}+\lambda_{3}L_{s}. \end{array}$$

Based on this objective function, we use the Adam Optimizer $\left(\beta_{1}=0.5,\beta_{2}=0.999\right)$ . The learning rate is set to 0.00001 , and the parameters $\lambda_{0}$ , $\lambda_{1}$ , $\lambda_{2}$ , and $\lambda_{3}$ are set to 2, 100, 20, and 100, respectively.

## 5. Experiments

### 5.1. Dataset Details

To compare our method with existing work, we used three real datasets, optical shadow removal (OSR) [5], Kligler’s [2], and Jung’s [3] datasets. Since these three datasets have different characteristics, as shown in Table 1, they are suitable to evaluate the robustness gains by learning on the proposed SynDocDS dataset. At this time, Bako’s dataset [1] and RDSRD [6] are unfortunately not publicly available. Images were resized to 512 × 512. As shown in Table 1, the OSR dataset [5] and Kligler’s dataset [2] have overlap in the documents used as the background, so we divided them into a training set, a validation set, and a test set so that they were not included in different sets. In addition, some datasets do not contain shadow masks. Therefore, we created new shadow masks by applying Otsu’s binarization method to the difference between shadow and shadow-free images.

**Real document image datasets.** The OSR dataset [5] comprises 237 triplets of shadow images, shadow-free images, and shadow masks. Among them, 163 triplets were for training, 28 for validation, and 46 for testing. Kligler’s dataset [2] contains 300 pairs of shadow images and shadow-free images from four categories: handwritten documents, printed documents, posters, and fonts. Among them, 192 triplets were for training, 59 for validation, and 49 for testing. Jung’s dataset [3] contains 87 pairs of shadow images and shadow-free images. To increase the testing set, we changed the original split by adding 10 pairs to the original testing set, resulting in 30 pairs for testing. Then, we randomly divided the remaining 57 pairs into training and validation sets containing 50 and 7 pairs, respectively.

**SDSRD.** As the SDSRD [6] is not publicly available with complete data, we rendered a nearly equal number of images using the provided python script and Blender [29]. In the original paper, 8309 triplets comprising shadow-free images, shadow images, and shadow mattes were generated, 7533 of which served as training data and the remaining 776 as test data. Therefore, we generated 8018 triplets using the same background document images as in [6], with 6995 as training data and the remaining 1025 as test data. These were split so as not to contain the same background image.

**SynDocDS dataset.** Additionally, we used the proposed SynDocDS dataset for training. The SynDocDS dataset comprises triplets of a shadow image, a shadow-free image, and a shadow matte. Although it is possible to create as many images as the number of material combinations, 50,000 quadruplets were created in advance for experiments, as noted by ^†^ in Table 1. Among them, 40,000 were for training, 5000 for validation, and 5000 for testing.

### 5.2. Compared Methods and Evaluation Metrics

**Models.** We compared the proposed network to six state-of-the-art methods, including three traditional methods by Bako et al. [1], Kligler et al. [2], and Jung et al. [3], along with three basic deep learning-based methods, STCGAN-BE [6,7], BEDSR-Net [6], and DHAN [9], which do not require particular input or mechanisms. Because the training codes of STCGAN and BEDSR-Net are not publicly available, we re-implemented their models according to their papers.

**Evaluation metrics.** To perform a quantitative comparison, we followed previous shadow removal approaches [7,9] and evaluated the root mean square error (RMSE) in the LAB color space. In addition, we reported the peak signal-to-noise ratio (PSNR) and structural similarity index measure (SSIM) to evaluate the quality of the shadow removal results. Finally, to evaluate the improvement in readability, we compared the performance of optical character recognition (OCR). These quantitative scores were reported as the average score over three training sessions.

### 5.3. Visual Quality

**Figure 7:**
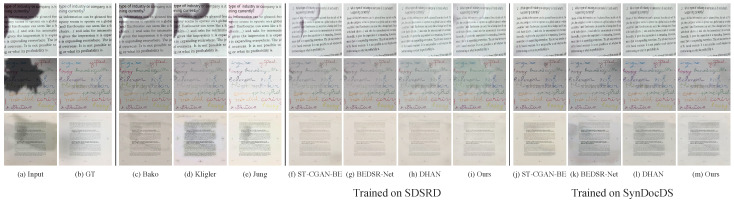
Qualitative comparison. From top to bottom, samples are from the OSR [5], Kligler’s [2], and Jung’s [3] datasets, respectively.

**Training on a synthetic dataset.** Here, we trained a deep learning-based network on the synthetic dataset, the SDSRD [6], and the proposed SynDocDS dataset. Then, we evaluated each model on real image datasets. Note that all data from the training, validation, and test sets were used for the evaluation, except for those used for the observation of shadows in document images. As shown in Table 3, the networks trained on our proposed SynDocDS dataset performed better than the SDSRD. Figure 7 illustrates the qualitative results for each method. Even without using real data, deep learning-based models removed shadows reliably. Furthermore, we found that the models trained on our SynDocDS dataset were more robust and provided a higher quality, indicating sufficient effectiveness in using deep learning models without real data. In addition, our DSFN gave better results with different datasets than other methods quantitatively and qualitatively.

**Training dataset comparison.** We compared each network on three learning patterns for deep learning-based methods to measure the effectiveness of the proposed SynDocDS dataset. The learning patterns were (i) only trained on a real dataset, (ii) only trained on a synthetic dataset, and (iii) pre-trained on a synthetic dataset and then fine-tuned on a real dataset. The networks were then evaluated with the test set of real datasets for all learning settings. As shown in Table 4, pre-training on the SynDocDS dataset significantly improved the quality of the results when fine-tuning was performed even on a limited number of real images.

### 5.4. Text Readability

To evaluate improvement in document readability, we compared OCR’s performance on the output images from the networks trained on the proposed SynDocDS dataset. We used images with detectable text in the OSR dataset [5], and images were center cropped to 512 × 512. First, we applied Tesseract [49], an open-source OCR tool, to recognize text in the ground truth image and output images. Next, we evaluated the performance of OCR by comparing the distances of text strings using the edit distance [50]. As shown in Table 5, the proposed DSFN produced the best results.

## 6. Discussion

### 6.1. Quantitative Score

It is noted that acknowledging that a simplistic comparison of scores does not provide a comprehensive evaluation is essential. The ground truth image does not perfectly represent the input image without shadows due to slight pixel deviations and variations in brightness and color caused by environmental factors during image capturing. These points have also been discussed in Le et al. [15]. Therefore, pixel-level error calculations such as RMSE and the PSNR may occur with qualitative superiority but quantitative inferiority. However, the SSIM, which is close to our visual perception, is possibly a more appropriate performance measure, and our network outperforms others on the SSIM. Relying on quantitative comparisons only can lead to overfitting of the dataset and is not advisable, specifically when the ground truth data are not flawless. Hence, a comprehensive evaluation considering qualitative evaluation, quantitative evaluation, and OCR performance that considers future applications is required. Our network consistently achieves superior results, as shown in Table 5 and the qualitative results in this paper and Appendix A.

### 6.2. Dataset Diversity

As shown in Table 4, the network trained on our proposed dataset shows a competitive or outperformed performance on most metrics as the network trained on real data, which is the same domain as the test data. Although the three datasets used in the evaluation have different characteristics, as shown in Table 1, we found that our proposed dataset is effective for those datasets. Hence, our SynDocDS dataset is diverse and provides robustness to deep shadow removal networks.

### 6.3. Limitations

As shown in Table 4, the network only trained on the SynDocDS dataset performed better on the OSR [5] and Kligler [2] datasets, while it performed worse on the Jung dataset [3] than the network trained on the real dataset in terms of the PSNR. There is a domain gap between Jung’s data and our data, indicating room for improvement in our dataset. Furthermore, our dataset has a large number of diverse samples, making the network training convergence time-consuming. Effective data selection is required to make our dataset productive for networks.

### 6.4. Future Works

The proposed synthetic dataset was shown to provide deep shadow removal networks with robustness to document images with shadows in various environments. The data creation pipeline demonstrated in this study can be applied to creating training datasets in various tasks, such as OCR, Document Rectification, and Layout Recognition. In the future, we would like to explore creating datasets adaptable to any task useful for such document analysis.

## 7. Conclusions

This paper introduces a dataset for document shadow removal, SynDocDS, and a novel shadow removal network, the DSFN. Simulating various environments through rendering software allows us to obtain a large, diverse dataset for training deep learning models. Furthermore, by observing the characteristics of the document images and augmenting the shadow diversity through physics-based shadow synthesis, we can generate shadows with various shadow attenuation characteristics that are more plausible as document images. We showed that deep neural networks trained on the proposed SynDocDS dataset alone were able to reliably remove shadows from real images and perform better than when training on existing synthetic data. Furthermore, using the SynDocDS dataset for pre-training, we obtained significantly better results with fine-tuning on a limited number of real images. Finally, through extensive experiments, we demonstrated that the proposed DSFN clearly outperforms other methods quantitatively and qualitatively.

## Figures and Tables

**Table 1:** Summary of document shadow removal datasets.

Dataset	#pairs	#documents	Characteristics of Images	Shadow Mask
Bako [1]	81	11	Light shadows, text only	-
Kligler [2]	300	25	Dark shadow, complex content	-
Jung [3]	87	87	Multicast shadows	-
OSR [5]	237	26	Colored shadows, text only	✓
RDSRD [6]	540	25	Complex content/shadows	✓
RDD [10]	4916	<500	Complex content/shadows	✓
SD7K [11]	7620	350	Complex content/shadows	✓
SDSRD [6]	8309	970	Synthetic shadows, diverse contents/shadows	✓
SynDocDS (ours)	50,000 ^†^	1420 ^†^	Synthetic documents and shadows, diverse textures/contents/shadows	✓

^†^ It is possible to create as many images as the number of material combinations.

**Table 2:** Property details of the document mesh.

Property		Value
Mass		0.4
Friction		15
Stiffness	Tension	80
	Compression	80
	Shear	80
	Bending	10
Damping	Tension	25
	Compression	25
	Shear	25
	Bending	1

**Table 3:** Quantitative comparison. The arrows indicate whether a high score (↑) or low error (↓) indicates better performance. The best and second-best results are marked in red and blue, respectively. The best score in each training dataset is **bold**.

Training Dataset	Method	Average		OSR Dataset [5]		Kliglers’s Dataset [2]		Jung’s Dataset [3]	
		**PSNR** (↑)	**SSIM** (↑)	**PSNR** (↑)	**SSIM** (↑)	**PSNR** (↑)	**SSIM** (↑)	**PSNR** (↑)	**SSIM** (↑)
-	Original	15.72	0.9100	17.25	0.9326	14.73	0.8874	14.93	0.9211
-	Bako [1]	22.31	0.9494	20.57	0.9599	24.78	0.9443	18.54	0.9383
	Kligler [2]	19.88	0.9184	18.17	0.9251	21.31	0.9179	19.62	0.9018
	Jung [3]	15.74	0.9260	15.36	0.944	13.72	0.9053	23.76	0.9483
SDSRD [6]	STCGAN-BE [6,7]	21.94	0.9355	19.22	0.9302	24.27	0.9438	21.20	0.9212
	BEDSRNet [6]	22.76	0.9459	19.24	0.9434	**25.72**	0.9524	22.14	0.9303
	DHAN [9]	20.28	0.9512	17.48	0.9473	21.68	0.9552	23.10	**0.9483**
	DSFN (Ours)	**23.00**	**0.9590**	**19.74**	**0.9581**	25.53	**0.9630**	**23.16**	0.9480
SynDocDS (Ours)	STCGAN-BE [6,7]	25.1	0.9637	**23.41**	0.9696	27.01	0.9617	23.13	0.9547
	BEDSRNet [6]	25.69	0.9656	22.95	0.9696	28.50	0.9649	**23.48**	0.9571
	DHAN [9]	25.51	0.9703	22.33	0.9734	29.21	0.9717	21.45	0.9572
	DSFN (ours)	**25.70**	**0.9708**	22.50	**0.9739**	**29.24**	**0.9723**	22.20	**0.9575**

**Table 4:** Quantitative comparison by changing the training dataset. The arrows indicate whether a high score (↑) or low error (↓) indicates better performance. The best and second-best results are marked in red and blue, respectively. The best score in each method is indicated by **the green box**. The best score in each method is **bold**.

Method	Training Dataset	OSR Dataset [5]			Kliglers’s Dataset [2]			Jung’s Dataset [3]		
		**RMSE** (↓)	**PSNR** (↑)	**SSIM** (↑)	**RMSE** (↓)	**PSNR** (↑)	**SSIM** (↑)	**RMSE** (↓)	**PSNR** (↑)	**SSIM** (↑)
Original	-	9.86	17.57	0.9249	11.61	14.76	0.9010	12.45	13.96	0.8813
BEDSRNet [6]	Real dataset	5.72	23.37	0.9251	3.64	27.77	0.9569	5.18	24.42	0.9144
	SDSRD [6]	7.68	19.19	0.9055	4.72	24.58	0.9587	6.93	20.07	0.8880
	SynDocDS	5.51	23.97	0.9520	3.73	28.31	0.9689	4.69	22.97	0.9332
	SDSRD [6] + FT	5.04	23.76	0.9448	3.13	29.25	0.9687	4.16	24.06	0.9205
	SynDocDS + FT	**4.67**	**25.68**	**0.9648**	**2.73**	**30.05**	**0.9745**	**3.68**	**25.01**	**0.9330**
DSFN (ours)	Real dataset	8.46	24.34	0.9676	3.69	25.85	0.9738	4.25	23.62	0.9303
	SDSRD [6]	5.85	21.74	0.9546	3.65	26.75	0.9731	5.1	21.89	0.9213
	SynDocDS	5.42	24.20	0.9705	2.51	30.57	**0.9809**	5.59	21.20	0.9317
	SDSRD [6] + FT	5.66	25.38	0.9724	2.14	30.83	0.9799	3.71	24.82	0.9356
	SynDocDS + FT	**5.39**	**25.77**	**0.9728**	**1.97**	**32.02**	0.9802	**3.56**	**25.02**	**0.9361**

**Table 5:** Average edit distances between the inputs and outputs. The down arrow (↓) indicates better performance.

Method	Original	STCGAN-BE	BEDSR-Net	DHAN	DSFN (Ours)
Edit distance (↓) @OSR dataset [5]	172.26	28.52	28.20	26.64	**25.14**

**Figure A1:**
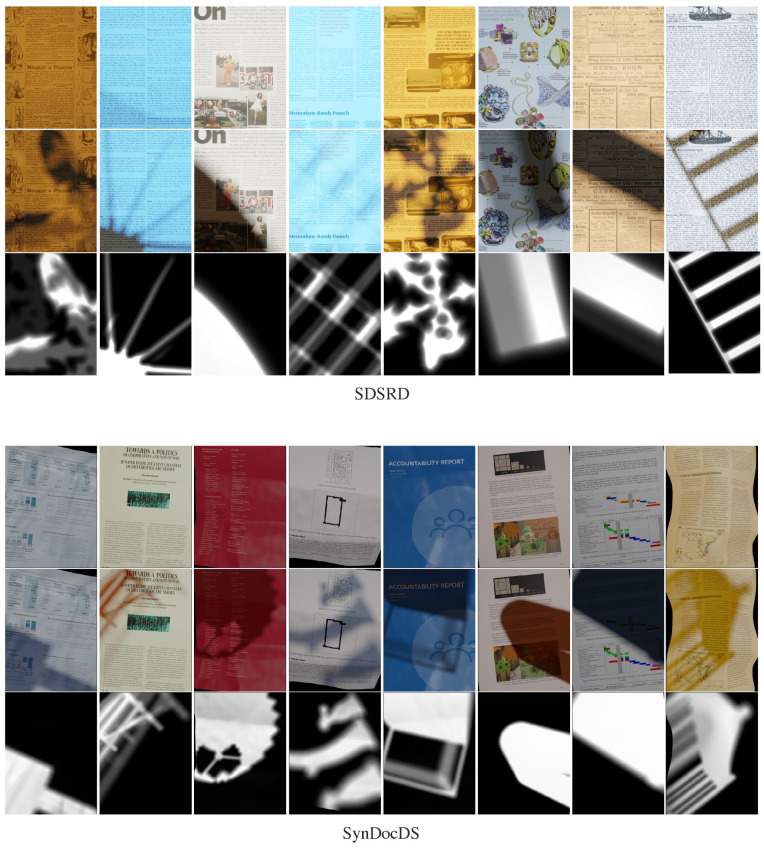
Example triplets of synthetic datasets. The top and bottom triplet samples are from the SDSRD [6] and SynDocDS dataset, respectively. From top to bottom: shadow-free images, shadow images, and shadow mattes.

**Figure A2:**
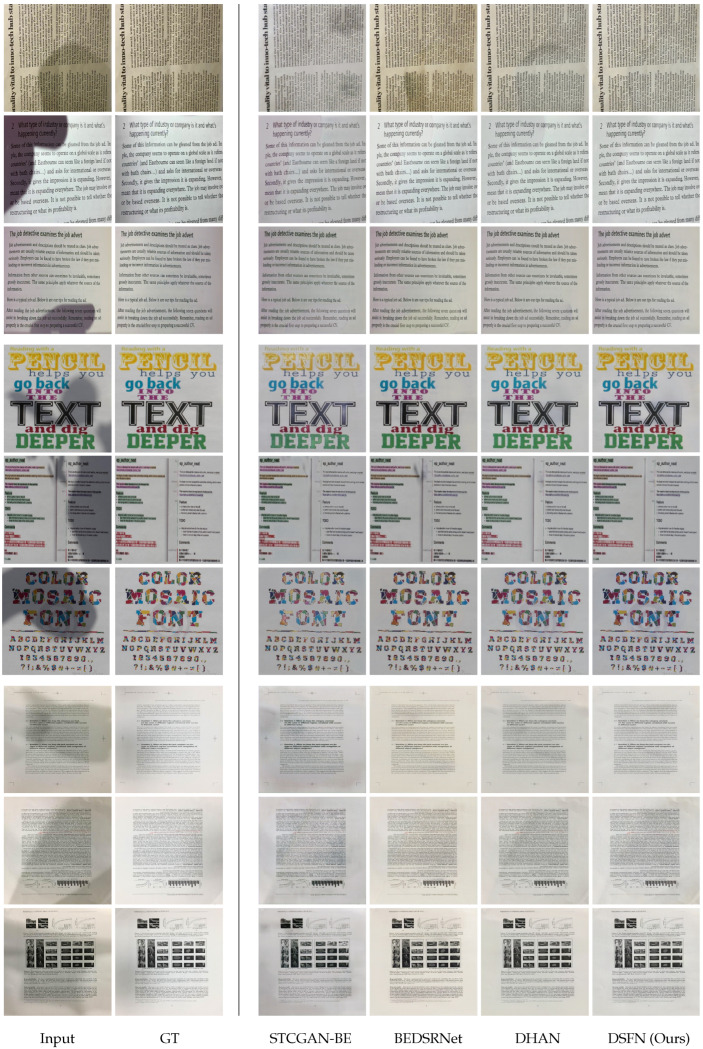
Qualitative comparison of each model trained on SynDocDS dataset. Results in the top, middle, and bottom three samples are from the OSR [5], Kligler’s [2], and Jung’s [3] datasets, respectively.

**Figure A3:**
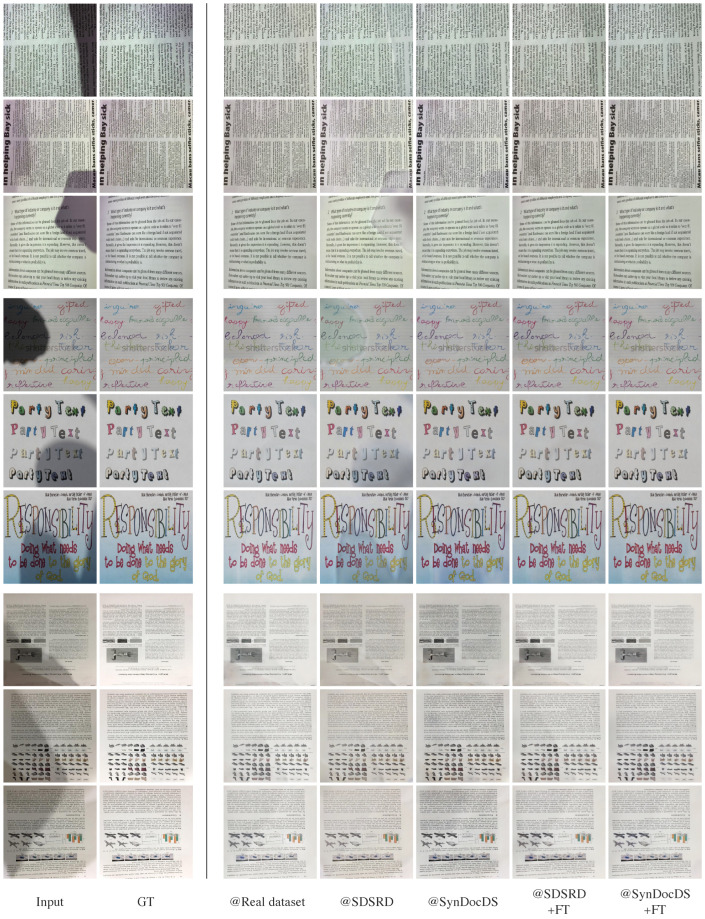
Qualitative comparison of our Dual Shadow Fusion Network (DSFN) trained on different datasets. The used training dataset is indicated by @. Results in the top, middle, and bottom three samples are from the OSR [5], Kligler’s [2], and Jung’s [3] datasets, respectively.

## Data Availability

The codes are available at this repository. Available online: https://github.com/ym4t50/SynDoc4DSFN (accessed on 19 October 2023).

## Appendix A

In this section, we show examples of synthetic datasets and results that could not be included in the main body of this paper due to space constraints. Figure A1 shows examples of the reproduced SDSRD [6] with codes provided as stated in Section 5 and our Synthetic Document with Diverse Shadows (SynDocDS) dataset. Figure A2 shows other qualitative results for each method corresponding to Figure 7 in the main paper. Furthermore, Figure A3 shows qualitative results corresponding to Table 4 in the main body of this paper.
